# Risk factors for acute human brucellosis in Ijara, north-eastern Kenya

**DOI:** 10.1371/journal.pntd.0008108

**Published:** 2020-04-01

**Authors:** Stella G. Kiambi, Eric M. Fèvre, Jared Omolo, Joseph Oundo, William A. de Glanville

**Affiliations:** 1 Ministry of Agriculture, Livestock and Fisheries, State Department of Veterinary Services, Nairobi, Kenya; 2 International Livestock Research Institute, Nairobi, Kenya; 3 Institute of Infection and Global Health, University of Liverpool, Liverpool, United Kingdom; 4 Kenya Field Epidemiology and Laboratory Training Program, Ministry of Health, Nairobi, Kenya; 5 Department of Infectious Disease Epidemiology, London School of Hygiene and Tropical Medicine, London, United Kingdom; 6 Institute of Biodiversity, Animal Health and Comparative Medicine, University of Glasgow, Glasgow, United Kingdom; Swiss Tropical and Public Health Institute, SWITZERLAND

## Abstract

Brucellosis is an important zoonotic disease globally, with particularly high burdens in pastoral settings. While the zoonotic transmission routes for *Brucella* spp. are well known, the relative importance of animal contact, food-handling and consumption practices can vary. Understanding the local epidemiology of human brucellosis is important for directing veterinary and public health interventions, as well as for informing clinical diagnostic decision making. We conducted a cross-sectional study in Ijara District Hospital, north-eastern Kenya. A total of 386 individuals seeking care and reporting symptoms of febrile illness were recruited in 2011. Samples were tested for the presence of *Brucella spp*. using a real-time PCR (RT-PCR) and results compared to those from the test for brucellosis used at Ijara District Hospital, the febrile Brucella plate agglutination test (FBAT). A questionnaire was administered to all participants and risk factors for brucellosis identified using logistic regression with an information theoretic (IT) approach and least absolute shrinkage and selection (LASSO). Sixty individuals were RT-PCR positive, resulting in a prevalence of probable brucellosis of 15.4% (95% CI 12.0–19.5). The IT and LASSO approaches both identified consuming purchased milk as strongly associated with elevated risk and boiling milk before consumption strongly associated with reduced risk. There was no evidence that livestock keepers were at different risk of brucellosis than non-livestock keepers. The FBAT had poor diagnostic performance when compared to RT-PCR, with an estimated sensitivity of 36.6% (95% CI 24.6–50.1) and specificity of 69.3% (95% CI 64.0–74.3). Brucellosis is an important cause of febrile illness in north-eastern Kenya. Promotion of pasteurisation of milk in the marketing chain and health messages encouraging the boiling of raw milk before consumption could be expected to lead to large reductions in the incidence of brucellosis in Ijara. This study supports the growing evidence that the FBAT performs very poorly in the diagnosis of brucellosis.

## Author summary

Brucellosis is a neglected, debilitating disease transmitted from livestock to people through direct contact with animal birth or abortion products or through the consumption of raw animal products, particularly milk. The febrile Brucella agglutination test (FBAT), a low-cost laboratory test for brucellosis, is widely used in health facilities throughout East Africa. Previous studies have shown this test has poor diagnostic specificity, contributing to high levels of overdiagnosis in low brucellosis prevalence settings. In this study, we compared the performance of the FBAT with real-time PCR in a high brucellosis prevalence setting in Ijara, Kenya. Our findings suggest very low FBAT diagnostic sensitivity and specificity, which could contribute to brucellosis cases being both missed and over-diagnosed in settings where the disease is common. The use of this test should therefore be phased out. Local epidemiological information can assist diagnostic decision making for brucellosis while awaiting replacement with more accurate tests. In the town of Ijara, at the time of the study, livestock ownership did not predict brucellosis risk. Rather, consumption of milk purchased from local shops and the market and not boiling milk before consumption were the most important predictors of acute disease. These findings also suggest public health interventions in the milk marketing chain could substantially reduce the incidence of human brucellosis.

## Introduction

Brucellosis is one of the oldest and most widely distributed zoonotic diseases [1]. It is caused by infection with intracellular gram-negative coccobacilli of the family *Brucellaceae*. The majority of cases of human disease have been associated with infection with *Brucella abortus* or *B*. *melitensis* [2], both of which circulate in wild and domestic ungulates. Livestock are almost always the source of human infection [3], which primarily occurs through direct or indirect contact with the birth or abortion products of infected animals or by consumption of contaminated, non-heat treated livestock products such as milk, meat, and blood [4–7]. The disease is an important occupational hazard for farmers and animal health workers [8,9]. However, in areas with inadequate veterinary public health provision, and where the consumption of raw animal products is common, the transmission of *Brucella* spp. via the food chain can be considered to represent a potential risk for livestock keepers and non-livestock keepers alike [10]. The prevalence of *Brucella* spp. infection in livestock is often highest in arid and semi-arid areas, and particularly where cattle, small ruminants and/or camels are reared in large herds or flocks under extensive management [1,11,12]. Control programmes that aim to reduce the human health impacts of *Brucella* spp. through interventions in the animal reservoir, such as vaccination, and/or through improved food safety have been reported to translate to substantial public health benefits in endemic areas [1,13,14].

The most common presentation of human brucellosis is a non-specific febrile illness, with clinical manifestations commonly including headache, malaise, body aches, and back and joint pain [7,15,16]. The disease can become chronic and may affect any organ system, resulting in severe illness such as osteomyelitis [16], epididymo-orchitis [17], neurologic disease [18], and cardiovascular complications [19]. Mortality can occur in untreated and severe cases [15]. Clinical detection and management of human brucellosis remains a major challenge in many of the countries in which the disease is endemic. The diagnosis of brucellosis cannot be made on the basis of clinical signs and symptoms alone, and definitive diagnosis relies on the detection of *Brucella* spp. by culture or molecular techniques or the demonstration of raised titres on paired acute and convalescent serological tests [20,21]. The capacity to perform such testing is often limited or not available in rural health facilities in the low- and middle-income countries where the burden of human brucellosis is highest [22]. In government health facilities in Kenya, the febrile Brucella plate agglutination test (FBAT), a rapid plate-based assay, has been the principal diagnostic test used for the diagnosis of human brucellosis. Recent research from a low brucellosis prevalence setting in western Kenya has shown that this test performs poorly, leading to misdiagnosis and inappropriate clinical management of people presenting with febrile illness [23]. Where imperfect diagnostic assays are being used, there is a need for baseline, locally-specific epidemiological information on human brucellosis that can inform pre- and post-test probabilities for the disease when interpreting laboratory results.

In this study, we identify individual-level exposures that explain variation in acute brucellosis risk among patients attending an outpatient facility at the Ijara District Hospital in north-eastern Kenya. By identifying risk factors for human disease, we aim to achieve two objectives. Firstly, to inform the development of public health interventions intended to reduce the incidence of human brucellosis and secondly, to contribute epidemiological information that can inform diagnostic decision making in individuals presenting to health facilities with febrile illness in north eastern Kenya. Recent work has suggested the prevalence of human brucellosis in Garissa County is likely to be high [11,24]. We therefore also aim to assess the performance of the FBAT in the diagnosis of acute human brucellosis in a high prevalence setting.

## Methods

### Study design and study site

**Fig 1:**
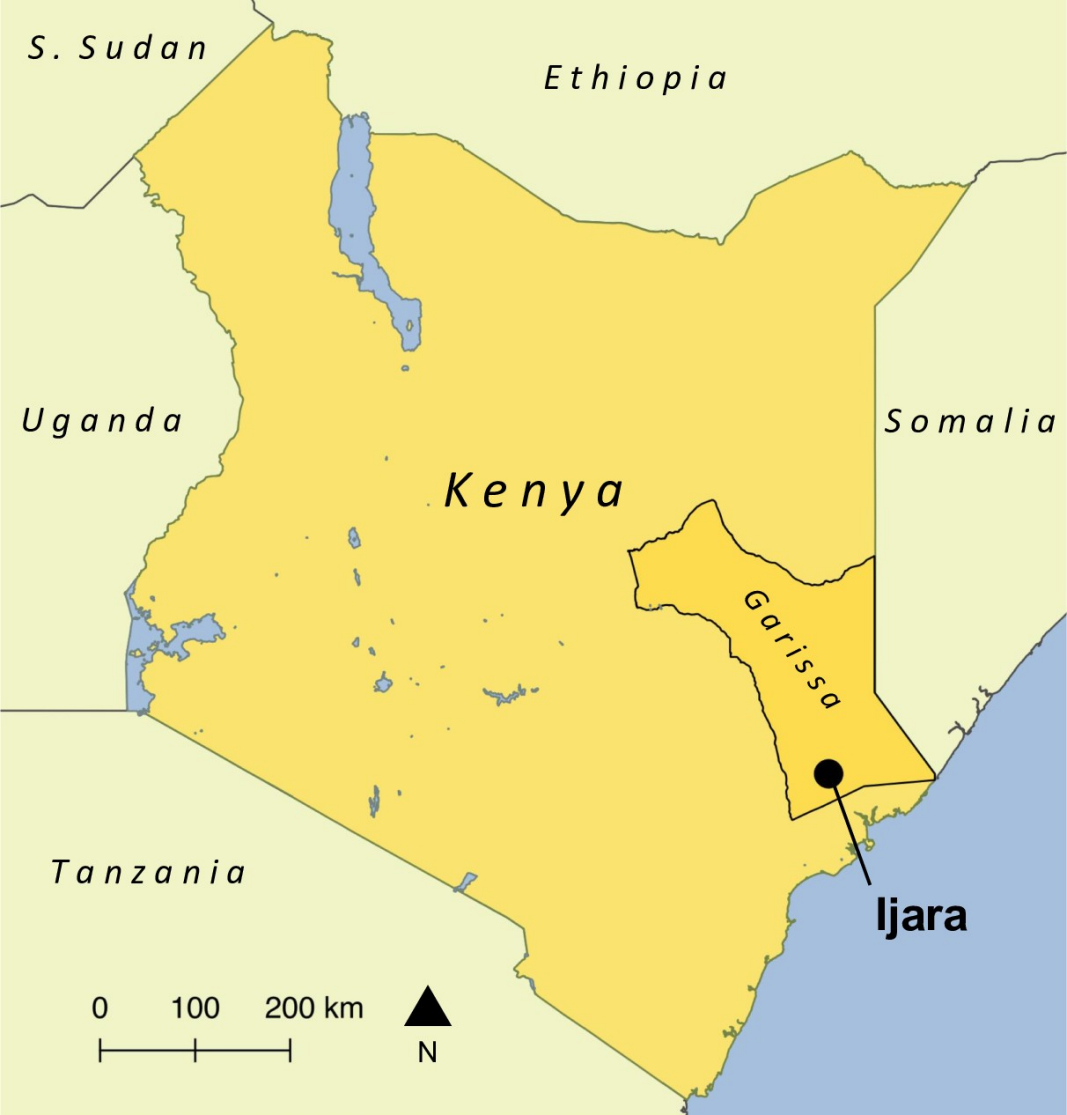
Location of the town of Ijara within Garissa County, Kenya. Map created using QGIS version 2.14.3. Base layers from GADM (https://gadm.org/).

This hospital-based cross-sectional study was conducted at the Ijara District Hospital in Garissa County. Garissa County is a semi-arid area in north eastern Kenya (Fig 1) which receives an average annual rainfall of 250 to 350mm [25]. Between 60 and 80% of people in the county are engaged in pastoral-based livelihoods centred around cattle, sheep, goat and camel production for both subsistence and commercial purposes [25]. According to the 2009 census, Ijara District had a population of 92,488 people [26]. Ijara District hospital is a 37-bed primary care hospital run by the Kenyan Ministry of Health [27].

### Sampling and data collection

Patients attending the outpatient clinic of Ijara Hospital from January to March 2011 were recruited. Patients aged 2 years and above reporting fever during their current illness were eligible for inclusion. A total of 386 participants were enrolled, with the sample size determined using standard formula for estimating a single proportion [28] and based on a desire to estimate the prevalence of brucellosis in the patient population with a maximum of 5% error at the 95% confidence interval. Review of hospital records indicated that around 40 febrile patients were seen at the outpatient clinic of the Ijara District hospital daily. To achieve a manageable target of 12 recruited cases per day, we sought to enrol every third eligible patient.

Three millilitres of blood were aseptically drawn into an additive free vacutainer by venipuncture from the cephalic or median cubital vein of each consenting and assenting participant. Blood samples were allowed to clot at room temperature and then centrifuged to obtain both serum and blood clots. Serum was tested for the presence of antibodies to smooth *Brucella* spp. using a febrile Brucella agglutination test (FBAT) (Febrile Serodiagnostics, Biosystems, Spain). This was the principal diagnostic assay for brucellosis at the participating health facility at the time of the study. The test was performed according to manufacturer’s instructions. Briefly, this involved placing two drops (~50ul) of serum on a white tile and mixing each with a drop of the rapid test reagent (“*abortus*” or “*melitensis*” antigen) and gently agitating on a shaker for two minutes while observing for agglutination. The presence of agglutination to either antigen reagent solution was considered to indicate a positive test. Blood clots were stored at –20°C before being transported to the Centers for Disease Control and Prevention Kenya (CDC-Kenya) laboratory in Nairobi for RT-PCR testing.

A structured, pre-tested questionnaire was administered to each participant at the time of sample collection to collect data on demographics and possible risk factors associated with brucellosis, including livestock ownership, livestock contact and regular milk consumption practices. Parents or guardians were asked to assist with answering questions when the participant was less than 18 years old.

### Molecular testing by PCR

DNA extraction was performed from blood clots using the QIAamp DNA Mini Kit (QIAgen Inc, Amsterdam, Netherlands) according to the manufacturer’s instructions. Real Time PCR (RT-PCR) assays were performed using the AgPath-ID One-Step RT-PCR Kit (ABI, Foster City, California) and gene specific primers for *Brucella spp*. The assays were performed on the ABI 7500 Fast RT-PCR instrument (ABI, Foster City, California). Each reaction mix included 50μM of gene specific forward and reverse primers, 10 μM of the gene specific probe, and 5μl of the DNA in a final reaction of 25 μl. The primer and probe sequences that were used in the assay were IS711 (F) GCTTGAAGCTTGCGGACAGT, IS711 (R) GGCCTACCGCTGCGAAT and IS711 (P) AAGCCAACACCCGGCCATTATGGT [29].

### Ethics statement

Approval to carry out the study was obtained from Kenya Medical Research Institute (KEMRI) Scientific Steering Committee (SSC number 1887) and National Ethical Review Committee (ERC). The study was also approved by the Board of Postgraduate Studies of Jomo Kenyatta University of Agriculture and Technology. Informed written consent or assent was obtained from all participants.

### Data analysis

The RT-PCR result was considered to be the reference test for assigning acute brucellosis status in this study. Potential predictors of RT-PCR positivity derived from the questionnaire were assessed using two approaches: 1) an information theoretic (IT) approach [30] and 2) Penalised regression using the least absolute shrinkage and selection operator (LASSO) [31].

### Information theoretic approach

The IT approach involved first defining a set of competing hypotheses (represented by one or more patient characteristics) that were considered as potential candidates in explaining variation in RT-PCR positivity. Logistic regression models testing these hypotheses were then developed and the model (and therefore hypothesis) that best explained variation in the outcome (RT-PCR positivity) was selected using minimal Bayesian information criterion (BIC). The BIC rewards goodness of fit while providing a penalty for the number of parameters estimated [32]. In developing the hypotheses to test, we were particularly interested in examining the role of livestock ownership in shaping variation in brucellosis risk, and to compare this to specific practices related to livestock ownership, such as assistance with parturition and source of milk consumed. We note that several of the available variables were naturally nested (e.g. livestock ownership and consumption of milk from own animals rather than an external source) or expected to be highly correlated (e.g. livestock ownership and assistance with animal birthing) and hypotheses were therefore developed to avoid including nested or naturally correlated variables in the same model [30]. We focus on livestock keeping in general as opposed to ownership of specific livestock species to reduce the complexity of specified hypotheses [33].

The hypotheses considered are listed in Table 1 and can be summarised as: livestock keeping with control for the potential confounding effect of age and sex provides the best model of RT-PCR positivity (M2); the effect of being a livestock keeper varies across different ages, with control for sex (M3); the effect of being a livestock keeper varies across different ages and sexes (M4); the effect of being a livestock keeper varies across different education levels with control for age and sex (M5); assisting with animal birthing (including reported contact with abortion and retained fetal membranes) with control for age and sex provides the best model of RT-PCR positivity (M6); consuming milk purchased from an external source (shop or market) and boiling milk before consumption with control for age and sex provides the best model of RT-PCR positivity (M7); the effect of consuming milk from an external source is different in those individuals who report boiling milk before consumption compared to those who do not, with control for age and sex (M8); consuming milk from an external source, boiling milk, and assisting with animal parturition with control for age and sex provide the best model of RT-PCR positivity (M9); boiling milk, regardless of source, with control for age and sex, provides the best model of RT-PCR positivity (M11); age and sex alone provide the best model of RT-PCR positivity (M12); being FBAT test positive provides the best model of RT-PCR positivity (M13). The intercept only model was also included for comparison (M1).

**Table 1:** Hypotheses considered in explaining variation in *Brucella* RT-PCR positivity.

Hypothesis	
M1	*Null*
M2	Livestock keeper + Age + Sex
M3	Livestock keeper x Age + Sex
M4	Livestock keeper x Age x Sex
M5	Livestock keeper x Education level + Age + Sex
M6	Assist with animal births + Age + Sex
M7	Milk from external source + Boil milk + Age + Sex
M8	Milk from external source x Boil milk + Age + Sex
M9	Milk from external source + Boil milk + Assist with animal births + Age + Sex
M10	Boil milk + Age + Sex
M11	Age + sex
M12	FBAT positive

After these hypotheses were developed, the IT analysis proceeded in two stages. First, univariable associations between each component variable and RT-PCR positivity were derived using logistic regression. Univariable logistic regression models were also developed to examine relationships between each component variable (i.e., being a livestock keeper and age, etc). To explore the potential for non-linear relationships between age and the log odds of RT-PCR positivity, we examined a range of specifications of age in years. These were i) age in quartile categories; ii) age in its linear form and standardised to have a mean of zero and standard deviation of 0.5; iii) age as a quadratic polynomial; and iv) age with non-linearities incorporated using restricted cubic splines with four knots. The specification of the variable age that resulted in the lowest BIC was used to test all hypotheses described in Table 1. Logistic regression models for this first step were run using base functions in the R statistical environment (version 3.4.2) with restricted cubic splines incorporated using the *rms* package [34]. The second step of the analysis was to fit the models representing the hypotheses shown in Table 1. Given the correlated nature of predictors in each hypothesis, we did not perform model averaging [32]. Instead, we made inference based on the top model selected using BIC. Logistic regression models for this second step were run within a Bayesian framework with weakly informative Cauchy prior on all co-efficients [35] using the *arm* package [36] in R.

### Least Absolute Shrinkage and Selection Operator

The food and animal exposures described in the hypotheses in Table 1 together with age and sex were included as potential predictors of RT-PCR positivity in the LASSO regression. Age was included in the form with lowest BIC (categorical, linear, quadratic or with splines, see above). Interaction terms were excluded. The LASSO model was fit in a Bayesian framework with a double exponential (Laplace) prior on all co-efficients [37]. The regression was run in JAGS via the *R2jags* package in R [38]. Convergence after a minimum burn-in of 50,000 and 100,000 iterations with a thinning interval of 10 was assessed by visual examination of MCMC chains. The output from LASSO was used to make predictions for the probability of acute brucellosis positivity on the basis of patient characteristics. Model predictive ability was assessed using the *c*-statistic [39].

### FBAT test performance

The diagnostic sensitivity, diagnostic specificity and positive and negative predictive values of the FBAT were calculated considering RT-PCR as the reference result.

## Results

### Participant characteristics

The median age of recruited participants was 26 years, with a range between 6 and 82 years. The majority of participants were female (n = 238, 61.7%). Most participants reported either having reached a maximum of primary school education (n = 155, 40.2%) or having no education (n = 130, 33.7%) (Table 2). Approximately half of all participants reported living in households where livestock were kept (n = 181, 46.9%), but a minority reported regularly assisting with animal births (n = 38, 9.8%). The majority of individuals reported regularly consuming milk from outside the household (purchased from a market and/or shop) (n = 288, 74.6%) and to not routinely boiling milk before consumption (n = 230, 59.6%). One hundred and seventy-eight individuals (46.1%) reported both regularly consuming milk from outside the household and to not routinely boiling milk. Sixty individuals were RT-PCR positive, resulting in an observed prevalence of acute brucellosis of 15.4% (95% Confidence Interval (95% CI 12.0–19.5)).

**Table 2:** Participant characteristics and univariable associations with *Brucella* RT-PCR positivity.

	% of population (n)	% PCR positive (n)	OR (95% CI)
**Age (quartiles)**			
6–19 years	30.8 (119)	13.4 (16)	*Baseline*
20–25 years	22.3 (86)	17.4 (15)	1.36 (0.63–2.93)
26–34 years	23.8 (92)	15.2 (14)	1.16 (0.53–2.50)
35–82 years	23.1 (89)	16.9 (15)	1.30 (0.61–2.80)
**Age (linear)**			
Age	-	-	1.09 (0.64–1.88)
**Age (quadratic)**			
Age	-	-	1.27 (0.87–1.87)
Age*Age	-	-	0.84 (0.66–1.07)
**Sex**			
Female	61.7 (238)	14.3 (34)	*Baseline*
Male	38.3 (148)	17.6 (26)	1.27 (0.73–2.23)
**Livestock keeper**			
No	53.1 (205)	18.0 (37)	*Baseline*
Yes	46.9 (181)	12.7 (23)	0.66 (0.37–1.16)
**Education**			
No Education	33.7 (130)	19.2 (25)	*Baseline*
Primary	40.2 (155)	11.6 (18)	0.55 (0.29–1.06)
Secondary	18.1 (70)	14.3 (10)	0.70 (0.31–1.56)
Tertiary and above	8.0 (31)	22.6 (7)	1.23 (0.47–3.16)
**Assistance with animal births**			
No	90.2 (348)	15.8 (55)	*Baseline*
Yes	9.8 (38)	13.2 (5)	0.81 (0.30–2.16)
**Milk from external source**			
No^1^	25.4 (98)	1.0 (1)	*Baseline*
Yes	74.6 (288)	20.5 (59)	25.0 (3.41–182.96)*
**Boil milk before consumption**			
No	59.6 (230)	23.4 (54)	*Baseline*
Yes	40.4 (156)	3.8 (6)	0.13 (0.05–0.31)*
**Rapid test positive**			
No	68.4 (264)	14.4 (38)	*Baseline*
Yes	31.6 (122)	18.0 (22)	1.31 (0.74–2.33)

^1^ These individuals all report consuming milk from their own animals.

### Risk factors

#### Univariable analysis

Univariable associations between each potential risk factor and RT-PCR positivity are shown in Table 2. The best fitting specification of age in terms of BIC was in its linear form, but there was no evidence for an association between increasing age and RT-PCR positivity (Table 2). Being a livestock keeper was associated with reduced odds of RT-PCR positivity (OR = 0.66), although the relationship was not significant at traditional levels (95% CI 0.37–1.16). The milk variable included three options: consume from own animals, purchase from market, and purchase from a shop. Both purchase from market and from a shop had large and similar odds ratios when compared to consume from own animals (25.5 and 18.2, respectively), and these two levels were combined. Consuming any purchased milk was associated with substantially elevated odds (OR = 25.0, 95% CI 3.41–18) while boiling milk before consumption was associated with substantially reduced odds (OR = 0.13, 95% CI 0.05–0.31) (Table 2).

Relationships between each of the predictors under consideration are presented in Table 3. Males were more likely than females to report being livestock keepers, assisting with animal births, and to have primary school education or above. Males were significantly less likely to consume milk from an external source, and to have positive FBAT test results. Individuals reporting to be from livestock keeping households were significantly more likely to assist with animal births than non-livestock keepers and significantly less likely to consume milk from an external source. They were also significantly less likely to have positive FBAT results. There was a significant negative association between assisting with animal births and consuming milk from an external source, and boiling milk before consumption and having a FBAT positive test. Education was significantly negatively associated with age. There was strong evidence that individuals consuming milk from an external source were more likely to be FBAT positive.

**Table 3:** Results from univariable logistic regression comparing risk factors for acute brucellosis.

	Male	Livestock keeper	Assist with animal births	Milk from external source	Boil milk before consumption	FBAT positive	Education^1^
Male	**-**						
Livestock keeper	**1.54** (1.02–2.34) *	**-**					
Assist with animal births	**3.1** (1.55–6.20) *	**8.21** (3.13–21.6) *	**-**				
Milk from external source	**0.62** (0.39–0.99) *	**-**	**0.42** (0.21–0.85) *	**-**			
Boil milk before consumption	1.01 (0.66–1.53)	0.81 (0.54–1.21)	1.37 (0.70–2.68)	0.7 (0.44–1.11)	**-**		
FBAT positive	**0.48** (0.30–0.77) *	**0.52** (0.34–0.81) *	1.47 (0.74–2.93)	**7.37** (3.44–15.77) *	**0.32** (0.19–0.51) *	**-**	
Education^1^	**3.68** (2.29–5.91) *	0.64 (0.40–1.01)	0.40 (0.15–1.05)	1.21 (0.71–2.07)	1.57 (0.99–2.48)	0.89 (0.54–1.45)	**-**
Age (in years)^2^	1.16 (0.94–1.42)	1.07 (0.72–1.60)	1.33 (0.98–1.78)	1.0 (0.79–1.26)	0.85 (0.69–1.05)	1.01 (0.82–1.25)	**0.55** (0.41–0.74) *

* 95% confidence intervals do not include 1 (p value <0.05)

^1^Binary: no education and primary school education or above

^2^Only a predictor (not an outcome). Scaled to have a mean of 1 and standard deviation of 0.5.

### Information theoretic approach

There was a clear top model, M7, which was almost 6 BIC points lower than all other models (Table 4). On the basis of this model, individuals consuming milk purchased outside the home had substantially elevated odds of RT-PCR positivity (OR = 19.2, 95% Credibility interval (CrI) 4.8–68.8), whilst individuals reporting boiling milk before consumption had substantially reduced odds of being PCR positive (OR = 0.13, 95% CrI 0.06–0.32) (Table 5). There was no evidence that male gender or age were associated with RT-PCR positivity. Outputs from all other models are provided in the supplementary materials.

**Table 4:** BIC support for each hypothesis explaining variation in *Brucella* RT-PCR positivity.

Hypothesis	BIC	ΔBIC
M7	300.6	0.0
M9	306.4	5.8
M8	306.6	6.0
M10	324.6	24.0
M1	339.5	38.9
M12	344.6	44.0
M11	350.6	50.0
M5	351.8	51.2
M2	353.1	52.5
M6	356.2	55.6
M3	358.7	58.2
M4	372.3	71.8

**Table 5:** Outputs from the model for *Brucella* RT-PCR positivity with the greatest BIC support.

Risk factor	OR	95% Credibility Interval
Milk from external source	19.20	4.81–68.80*
Boil milk	0.13	0.06–0.32*
Age	1.02	0.59–1.59
Male	1.59	0.85–2.92

* 95% credibility intervals do not include 1

### Least Absolute Shrinkage and Selection Operator approach

The results from the LASSO regression are shown in Table 6. These results support the importance of the hypothesis selected using the information theoretic approach, with milk from an external source associated with substantially increased odds of RT-PCR positivity and boiling milk associated with substantially reduced odds. The 95% credibility intervals for the co-efficient for the effect of being a male included zero, but there was minimal shrinkage of this variable. All other variables were shrunken towards zero, reflecting their relative lack of explanatory power. Model co-efficients can be interpreted in terms of an average posterior predicted probability of acute brucellosis in a male participant in our study who reports consuming milk from an external source and not boiling milk before consumption of around 0.33 (95% CrI 0.23–0.45) compared to around 0.01 (95% CrI 0.001–0.03) in a male participant in our study who reports not consuming milk from an external source (i.e. only from own animals) and boiling milk before consumption. The *c*-statistic was 0.79, suggesting reasonable model predictive performance.

**Table 6:** Mean posterior co-efficient estimates and 95% credibility intervals (CrI) derived from LASSO analysis for relationship between *Brucella* RT-PCR positivity and participant characteristics.

	Co-efficient (95% CrI)
Intercept	-3.76 (-5.88, -2.24)
Milk from external source	2.65 (1.19, 4.71) *
Boil milk	-1.86 (-2.78, -1.04) *
Male	0.35 (-0.20, 0.97)
Age	0.03 (-0.51, 0.57)
Livestock keeper	0.02 (-0.52, 0.59)
Assist with animal births	0.08 (-0.82, 0.99)
Primary education or above	0.08 (-0.52, 0.71)

*95% Credibility intervals do not include zero

### FBAT test performance

The comparison of FBAT and RT-PCR results is shown in Table 7. The proportion of individuals with positive FBAT was 31.6% (95% CI 27.2–36.7). The sensitivity of the FBAT was 36.7% (95% CI 24.6–50.1) and specificity was 69.3% (95% CI 64.0–74.3). Using RT-PCR results as the reference standard, the FBAT diagnosed 38 false negatives and 100 false positives (Table 2). The positive predictive value in the study population was estimated as 18.0% (95% CI 11.9–26.3) and the negative predictive value was 85.6% (95% CI 80.7–89.5).

**Table 7:** Comparison of results from FBAT and *Brucella* RT-PCR testing.

		PCR		
		Positive	Negative	Total
**FBAT**	Positive	22	100	122
	Negative	38	226	264
	Total	60	326	386

## Discussion

We have explored a range of predictors for human brucellosis in individuals attending a district hospital in Ijara, north-eastern Kenya. Importantly, we were not able to find evidence for livestock ownership alone being a risk factor for acute brucellosis in this community. Public health measures to reduce the incidence of brucellosis therefore need to consider both livestock keepers and non-livestock keepers in their scope. Clinicians in the study area should also consider brucellosis in the differential diagnosis for individuals presenting with febrile illness regardless of livestock ownership status. Rather, milk consumption practices appear to be a major determinant of brucellosis risk in the study population.

We show that consuming milk purchased outside the household is associated with a substantially increased risk of acute brucellosis. To our knowledge, little has been published about the milk marketing chain in Ijara. However, in the local market, we observed that milk from multiple animals and herds was commonly aggregated into containers of 5 to 20 litres before sale. Hence, milk from a single brucellosis infected animal has the potential to contaminate the pooled milk from multiple uninfected animals. This risk amplification effect through bulk milk contamination is particularly relevant for *B*. *abortus* and *B*. *melitensis*, since these pathogens have a low human infectious dose [40]. Levels of *Brucella* spp. shedding in the milk of infected animals can be highly heterogeneous, with a majority of infected individuals shedding low levels intermittently and a minority shedding high levels regularly, so called ‘super-spreaders’ [41,42]. The pooling of milk from multiple sources increases the likelihood that these super-spreaders can contribute to bulk milk, and therefore the likelihood of *Brucella* spp. contamination in milk available for purchase. A study in Kampala, Uganda predicted a 47% reduction in risk of human brucellosis if pasteurisation was incorporated into the milk production chain [43]. Our own findings strongly suggest that the implementation of pathogen reduction steps in the milk marketing chain, such as the pasteurisation of all milk, could substantially reduce the incidence of human brucellosis in Ijara. We also show that boiling milk before consumption was strongly protective against brucellosis. This supports the findings of a number of other studies in East Africa showing either protective effects of boiling milk or increased risks with consuming it raw [8,11,24,44]. Health education messages that encourage safe milk preparation in the home could therefore also be expected to lead to a reduction in the incidence of brucellosis in Ijara. It is important to note that milk products are a major source of nutrition in the study area [25], and therefore any interventions should aim to achieve safe milk consumption rather than milk avoidance.

The IS711 RT-PCR used in this study is reported to be both highly sensitive and specific for the detection of *B*. *melitensis* and *B*. *abortus* [29]. However, studies have shown that individuals treated for and cured of brucellosis, without evidence of chronic infection or relapse, can be persistently PCR-positive for long periods of time [45], potentially influencing the specificity of PCR for diagnosis of acute clinical cases. In the absence of culture confirmation or paired acute- and convalescent-phase antibody levels, the RT-PCR positive participants that were used as reference cases in this study should be considered as ‘probable’ rather than ‘confirmed’ cases of brucellosis [46]. However, even with some uncertainty about the number of true cases, the observed prevalence of 15.4% RT-PCR positivity in this patient group suggests brucellosis is likely to be an important cause of febrile illness in patients attending the outpatient clinic of Ijara Hospital. This prevalence estimate is substantially higher than that from the limited number of studies in sub-Saharan Africa in which the results of tests done on individuals with clinical suspicion of brucellosis could be considered to meet case definitions for confirmed brucellosis. These include a prevalence of 3.5% in northern Tanzania [47] and 4.3% in south western Uganda [48]. Further work is needed in north eastern Kenya to understand the true contribution of human brucellosis to the aetiology and burden of febrile illness, including the incidence of infection in the general population. Our findings, and recently published results reporting a similar prevalence in nearby Garissa Provincial Hospital and Wajir County Hospital [24], suggest this contribution is likely to be large.

Given the probable rather than confirmed status of brucellosis cases in this study, the direct comparison of FBAT to PCR positives for test assessment should also be interpreted with caution. However, our findings support those from a previous study in western Kenya that demonstrated very poor performance of the FBAT in the diagnosis of human brucellosis [23]. Here, we find both poor sensitivity and specificity of this test, and very low positive predictive value in the population under study. In the absence of confirmatory testing (which was not performed in the participating hospital at the time of the study), low positive predictive value can be expected to contribute to high rates of overdiagnosis, which may substantially impact the health and economic resources of patients if they undergo unnecessary brucellosis treatment, which involves protracted courses of multiple antibiotics [49]. The moderately low negative predictive value of the FBAT in this high prevalence setting also suggests true positive cases were likely to be missed. This may increase the likelihood of progression from acute to chronic disease. While awaiting the establishment of better-performing diagnostic tests for human brucellosis in Kenya, clinicians should use their clinical judgment before ordering the FBAT and in their interpretation of any FBAT results obtained. Our risk factor findings suggest that, at the time of the study, individuals consuming milk purchased from the milk marketing chain in Ijara and those people who do not boil milk before consumption are at particularly elevated risk of acute brucellosis.

In addition to the absence of confirmed brucellosis cases in this study, additional study limitations need to be considered when interpreting results. The sample size was powered to estimate prevalence, rather than for a risk factor analysis. Whilst we were able to detect strong effects for source of milk and boiling milk using both IT and LASSO approaches, we found no evidence for a relationship between assistance with animal birthing (which included reported contact with abortion and fetal membrane products). Large quantities of *Brucella spp*. can be shed in birth products of infected animals [9] and contact with these products has been reported as being important for brucellosis transmission in other settings in East Africa [11,44]. It is important to note that this exposure was relatively rare in this population, and therefore a failure to detect an effect here should not be taken as evidence for a lack of transmission of *Brucella* spp. via this route, or through other routes that involve close contact with livestock or their products. Moreover, Ijara is an urban centre, and while Ijara District Hospital serves pastoralists from surrounding rural areas, the frequency of high-risk animal contact, and therefore the relative importance of animal contact as a transmission route compared to milk consumption practices, could be expected to be higher in rural areas with larger livestock populations. Further studies to examine the differences in the epidemiology of human brucellosis between urban and rural settings in the region would be valuable. In this study we have described the risks associated with milk consumption broadly, rather than milk consumption from a particular species. We did not record the source of milk purchased in markets or shops, but camel and cattle milk are commonly consumed in Garissa, and goat milk more rarely [25]. We also did not record household-level livestock production system or explore relationships between ownership of different livestock species and acute brucellosis risk. Further study is therefore required to understand the relative importance of different livestock species as reservoirs for *Brucella* spp. in Garissa County, which would be particularly important if livestock focused interventions are planned. Our findings provide further evidence to support the phasing out of the FBAT as a point of care diagnostic test for brucellosis [23]. The RT-PCR approach used in this study requires high levels of laboratory capacity and is not currently routinely available for use in primary and secondary health facilities in Kenya. There is therefore a great need to identify alternative point of care diagnostic tests for brucellosis that can replace the FBAT wherever it is currently being used. We did not evaluate its performance here, but the Rose Bengal test has been suggested as a possible replacement for the FBAT in low resource settings [23,50].

### Conclusion

Human brucellosis is likely to be an important cause of febrile disease in Ijara, north eastern Kenya, with transmission via the milk marketing chain particularly important. Public health interventions to improve the safety of unprocessed milk purchased from retailers in markets and the education of consumers on the benefits of consuming milk that has been adequately heat treated are needed. Such interventions could be expected to lead to reductions the incidence of human brucellosis. In addition to highlighting aspects of the epidemiology of human brucellosis in Ijara, we also provide further evidence to demonstrate that the FBAT, a laboratory test used throughout East Africa at the time of the study, has both poor sensitivity and poor specificity, and is likely to contribute to the mismanagement of febrile patients. Future studies should identify point of care tests that can replace the FBAT in brucellosis endemic settings.

## Supporting information

S1 File STROBE checklist. (PDF)

S2 File Results from all candidate models from information theoretic approach. (PDF)
