# Supplementary material for: Risk factors for acute human brucellosis in Ijara, north-eastern Kenya
Source: PLoS Negl Trop Dis. 2020 Apr 1;14(4):e0008108. doi: 10.1371/journal.pntd.0008108 (PMC7153889; doi:10.1371/journal.pntd.0008108)
Supplement: S2 File — (PDF) [file pntd.0008108.s002.pdf]

Supporting Information 2

"Milk consumption practices predict acute human brucellosis risk in Ijara, north-eastern Kenya"

Stella G. Kiambi, Eric M. Fèvre, Jared Omolo, Joseph Oundo, William A. de Glanville

Outputs from all candidate models in the information theoretic approach are shown in Table S1.

Table S1. Outputs from all candidate models explaining variation in *Brucella* RT-PCR positivity

Model/Hypothesis	OR	95% Credibility Interval
**M2**		
Livestock keeper	0.57	0.33 – 1.02
Age	1.08	0.70 – 1.71
Sex	1.39	0.82 – 2.68
**M3**		
Livestock keeper	0.63	0.37 – 1.05
Age	1.13	0.52 – 2.27
Sex	1.38	0.79 – 2.47
Livestock keeper x age	0.77	0.27 – 2.56
**M4**		
Livestock keeper	0.86	0.43 – 1.71
Age	1.73	0.65 – 4.69
Sex	1.85	0.94 – 3.53
Livestock keeper x Age	0.74	0.17 – 3.27
Livestock keeper x Sex	0.42	0.14 – 1.28
Sex x Age	0.51	0.15 – 2.78
Livestock keeper x Sex x Age	0.90	0.09 – 5.00
**M5**		
Livestock keeper	0.70	0.39 – 1.22
Education level	1.33	0.64 – 3.23
Age	1.06	0.63 – 1.73
Sex	1.35	0.81 – 2.42
Livestock keeper x Education level	0.41	0.13 – 1.57
**M6**		
Assist with animal births	0.71	0.30 – 1.60
Age	1.05	0.63 – 1.69
Sex	1.35	0.74 – 2.29
**M7**		
Milk from external source	**19.20**	**4.8 - 68.8**
Boil milk	**0.13**	**0.06 - 0.32**
Age	1.02	0.59 - 1.59
Male	1.59	0.85 – 2.92

**M8**		
Milk from external source	**19.76**	**3.43 – 84.20**
Boil milk	**0.20**	**0.02 – 1.89**
Age	1.07	0.60 – 1.96
Male	1.65	1.04 – 3.03
Milk from external source x Boil milk	0.70	0.06 – 10.66
**M9**		
Milk from external source	**19.3**	**3.44 – 85.11**
Boil milk	**0.14**	**0.06 – 0.30**
Assist with animal births	1.28	0.51 – 3.25
Age	1.03	0.61 – 1.76
Sex	1.59	0.85 – 2.77
**M10**		
Boil milk	**0.13**	**0.05 – 0.31**
Age	0.98	0.62 – 1.75
Sex	1.31	0.84 – 2.08
**M11**		
Age	1.05	0.71 – 1.60
Sex	1.34	0.76 – 2.09
**M12**		
Rapid test positive	1.37	0.78 – 2.17
